# Early Cellular Responses of *Neurospora crassa* to Azole Stress: Osmotic Adjustment, Redox Buffering, Sterol-Pathway Feedback, and Cell Wall Remodelling

**DOI:** 10.3390/antibiotics15080811

**Published:** 2026-08-19

**Authors:** Tomáš Pagáč, Ján Víglaš, Petra Olejníková

**Affiliations:** Institute of Biochemistry and Microbiology, Faculty of Food and Chemical Technology, Slovak University of Technology in Bratislava, Radlinského 9, 812 37 Bratislava, Slovakia; jan.viglas@stuba.sk

**Keywords:** *Neurospora crassa*, azole antifungals, early stress response, osmoregulation, redox buffering, glycerol, oxidative stress, cell-wall remodelling, chitin synthases, ergosterol

## Abstract

**Background/Objectives:** Azole resistance in fungal pathogens is a growing clinical and environmental concern. However, short-term cellular responses during the first hours of azole exposure remain insufficiently characterised, particularly in filamentous fungi. This study used *Neurospora crassa* as a genetically tractable model to investigate early cellular responses to azole stress. **Methods:** Exponentially growing mycelia were exposed to conidium-derived MIC_80_ reference concentrations of fluconazole, voriconazole, ravuconazole, and ketoconazole. Early responses were assessed by monitoring radial growth, intracellular glycerol, H_2_DCF-DA microscopy, antioxidant enzyme activities, Calcofluor White staining, and RT-qPCR analysis of genes associated with osmoregulation, sterol homeostasis, oxidative stress, and cell-wall remodelling. **Results:** Azole exposure was associated with reduced net post-transfer radial growth and rapid treatment- and time-dependent changes in intracellular glycerol. Induction of *hog1* together with treatment-dependent changes in *gpd1* and glycerol accumulation was consistent with an early osmoregulatory response. Sterol-homeostasis genes showed selective feedback regulation, and several cell-wall-remodelling genes underwent treatment-dependent transcriptional changes. Qualitative H_2_DCF-DA microscopy showed limited oxidant-associated fluorescence during azole challenge. Catalase activity remained close to control levels, whereas representative SOD-activity experiments showed larger fold changes after voriconazole and ravuconazole exposure. Representative Calcofluor White micrographs showed irregular septation and localised regions of enhanced cell-wall-associated staining. **Conclusions:** *Neurospora crassa* shows multiple concurrent early responses to azole stress involving osmotic adjustment, sterol-pathway feedback, antioxidant responses, and cell-wall remodelling. These findings provide a framework for future studies examining how such stress responses contribute to recovery, tolerance, or longer-term adaptation.

## 1. Introduction

Fungal infections pose an increasing threat to human health, agriculture, and food security [1,2]. Although they are generally less common than bacterial infections, fungal diseases are difficult to treat because fungi are eukaryotic organisms and therefore share many cellular features with their hosts. This phylogenetic proximity limits the number of selective antifungal targets and contributes to the relatively narrow therapeutic arsenal currently available. Recent public health assessments have also emphasised the clinical and environmental importance of azole-resistant *Aspergillus* spp., particularly in relation to the widespread use of azole fungicides outside human medicine [3].

Resistance, however, represents only one endpoint of fungal survival under sustained drug pressure. During initial antifungal exposure, fungal cells can mount transient stress responses that include growth restriction, osmolyte accumulation, metabolic rewiring, altered membrane homeostasis, antioxidant responses, and remodelling of the cell surface. These short-term responses are distinct from stable resistance and may influence subsequent survival trajectories [4,5].

Azole antifungals are particularly relevant in this context because they are widely used in both clinical and agricultural settings. Their primary mode of action is the inhibition of lanosterol 14α-demethylase (CYP51), a key enzyme in ergosterol biosynthesis. Disruption of ergosterol homeostasis compromises membrane function and impairs fungal growth, but fungi can respond by activating compensatory gene-expression programmes mediated by stress-responsive signalling pathways [6,7,8].

Several conserved pathways participate in fungal responses to antifungal stress, including the high-osmolarity glycerol (HOG) pathway, the cell wall integrity (CWI) pathway, calcium-dependent cascades, and TOR-related signalling networks [9,10]. In addition to sterol-pathway feedback, azole exposure may induce xenobiotic detoxification, antioxidant responses, and cell-wall biosynthetic pathways, reflecting the multifactorial nature of fungal stress responses to these drugs [11].

Our previous study [11] examined *Neurospora crassa* after a longer azole-exposure interval and characterised a broader response involving xenobiotic detoxification, including CYP65/ABCC-associated mechanisms, together with chitin synthesis and related cell-wall changes. The present study addresses a distinct temporal question by focusing on the first 1–6 h of exposure and examining glycerol accumulation and osmoregulatory gene expression, early sterol-homeostasis feedback, redox-associated responses, and an expanded set of genes involved in cell-wall synthesis and remodelling. CYP65 and ABCC detoxification genes were not re-examined because their involvement during the later response had already been characterised [11]. The early cell-wall changes observed here are considered in relation to the later chitin-associated phenotype reported previously, while recognising that the two studies used independent experimental datasets and do not establish a direct temporal sequence within the same cultures. Accordingly, the present findings are described as early cellular responses and are not interpreted as evidence of experimentally demonstrated post-exposure recovery, tolerance, or stable resistance.

The model filamentous fungus *Neurospora crassa* provides a useful system for dissecting fungal stress responses. Its well-characterised genetics and comparatively streamlined gene repertoire allow conserved stress-response mechanisms to be examined with fewer confounding factors than in many pathogenic fungi [12]. In this study, we focused on the first 1–6 h after azole exposure during exponential growth. We analysed radial-growth kinetics, intracellular glycerol accumulation, oxidant-associated fluorescence, antioxidant enzyme activity, Calcofluor White staining patterns, and transcriptional changes in genes related to osmoregulation, oxidative stress, sterol metabolism, and cell-wall remodelling.

## 2. Results

### 2.1. Early Changes in Relative Colony Growth and Time-Dependent Glycerol Accumulation upon Azole Exposure

To investigate the early response of *Neurospora crassa* to azole antifungals, exponentially growing mycelia were exposed to the previously determined conidium-based MIC_80_ concentrations of fluconazole (FLC; 15 µg/mL), voriconazole (VRC; 0.3 µg/mL), ravuconazole (RVC; 0.6 µg/mL), and ketoconazole (KTC; 0.4 µg/mL). These concentrations were used as standardised reference exposure conditions based on our previous susceptibility study [11].

**Figure 1:**
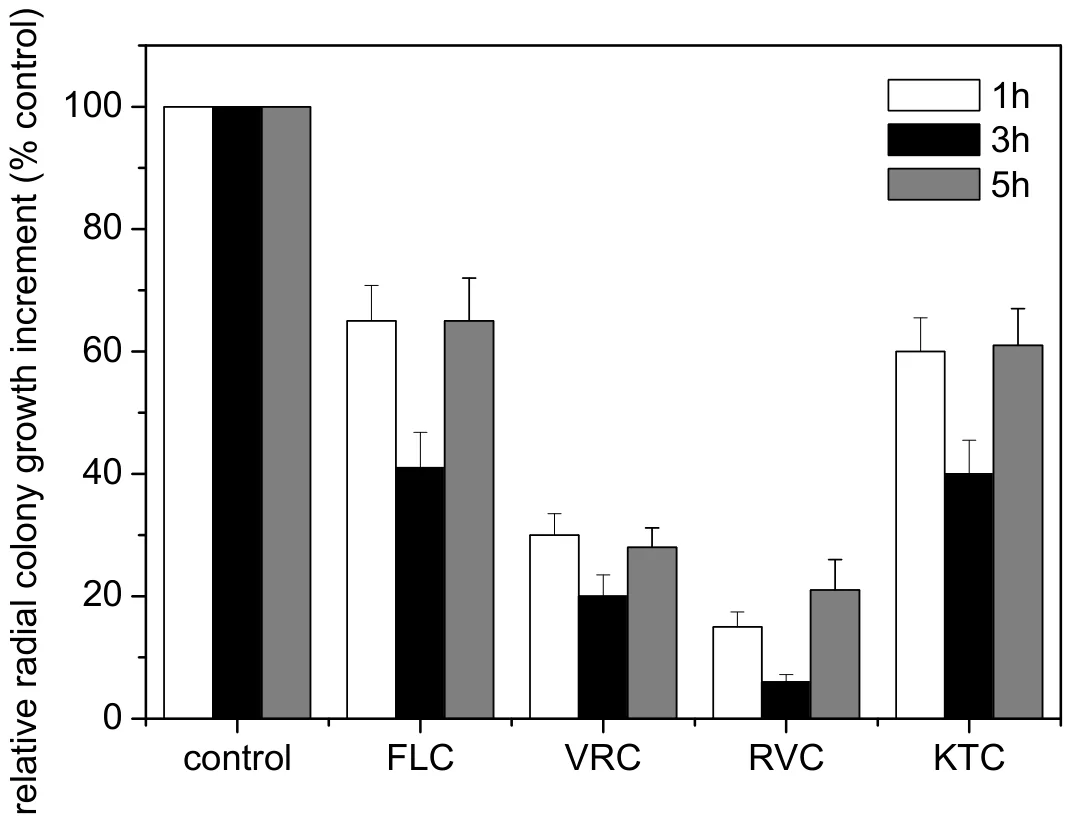
Net post-transfer radial growth of *Neurospora crassa* during early azole exposure. Colony diameters were measured immediately before transfer (D_0_) and after 1, 3, and 5 h (D_t_). Net growth was calculated as D_t_ − D_0_ and expressed relative to the corresponding increment of the matched vehicle control. Treatments were fluconazole (FLC), voriconazole (VRC), ravuconazole (RVC), and ketoconazole (KTC). Values are mean ± standard deviation from triplicate measurements and are presented descriptively.

Net post-transfer radial growth (D_t_ − D_0_), normalised to the corresponding matched vehicle-control increment, was lower under all azole treatments (Figure 1). The largest numerical differences were observed at 3 h, whereas relative values were higher at 5 h under several treatment conditions. These descriptive measurements do not demonstrate post-exposure recovery, tolerance, or stable adaptation.

**Figure 2:**
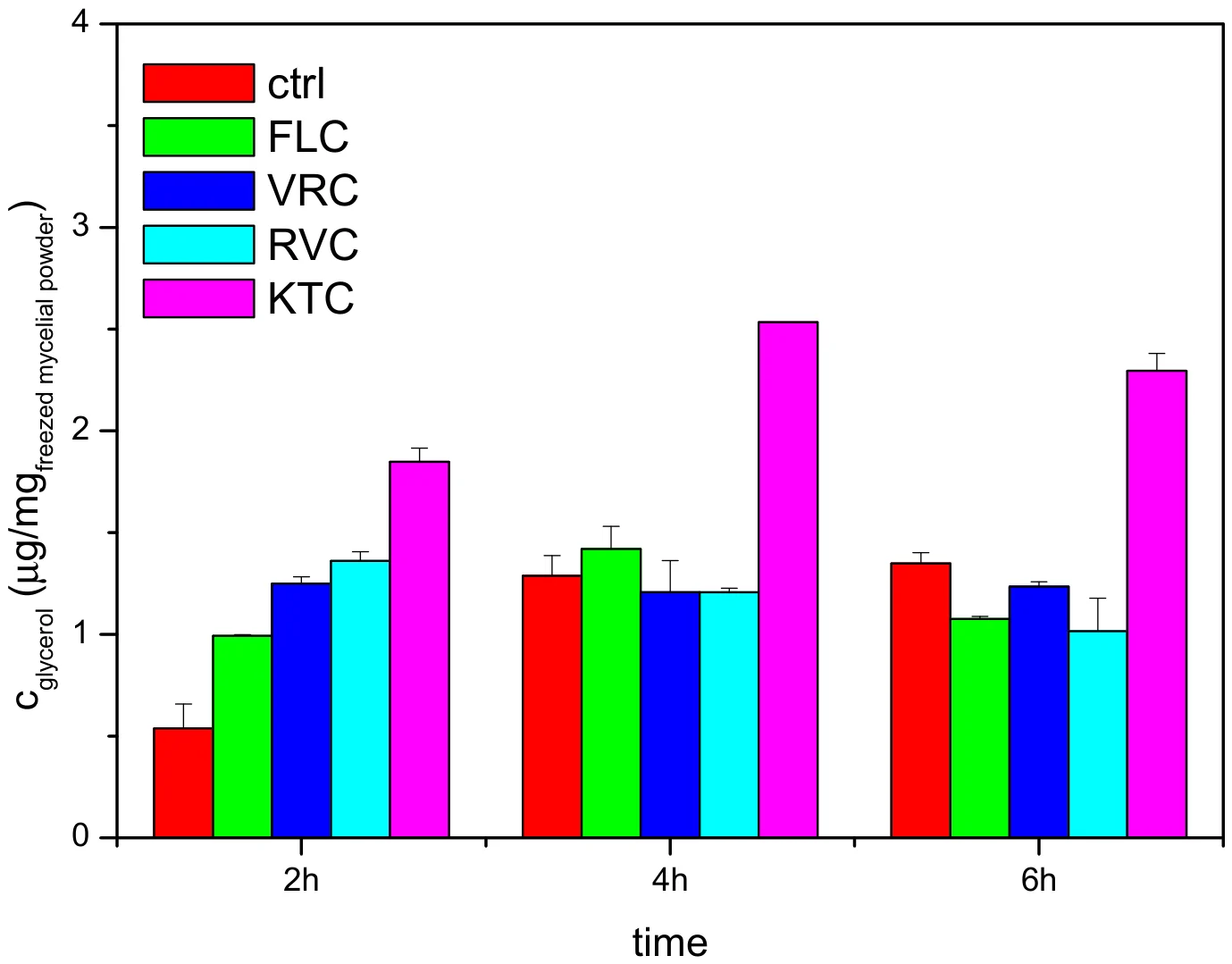
Time-dependent intracellular glycerol content in *Neurospora crassa* during early exposure to fluconazole (FLC), voriconazole (VRC), ravuconazole (RVC), or ketoconazole (KTC). Glycerol content is expressed as µg glycerol per mg frozen mycelial powder. Data shown are the mean ± standard deviation of three technical replicates from one representative biological experiment. Inferential analysis was performed on data from three independent biological replicates using two-way ANOVA with treatment and exposure time as factors, followed by Dunnett-adjusted treatment-versus-matched-control comparisons at each time point. Exact adjusted *p*-values are reported in Supplementary Table S5.

Intracellular glycerol was quantified after 2, 4, and 6 h of exposure (Figure 2). Two-way ANOVA detected significant effects of treatment (*p* = 3.22 × 10^−11^), exposure time (*p* = 3.66 × 10^−6^), and their interaction (*p* = 3.73 × 10^−6^). At 2 h, glycerol content was higher than the matched vehicle control after all four azoles (Dunnett-adjusted *p* = 0.00219 for FLC, 0.000132 for VRC, 0.0000733 for RVC, and 0.0000147 for KTC). At 4 h, only KTC remained higher than the control (*p* = 0.000354). At 6 h, FLC and RVC were lower than the control (*p* = 0.0140 and 0.0380, respectively), KTC remained higher (*p* = 0.00979), and VRC did not differ significantly (*p* = 0.281). Exact results are provided in Supplementary Table S5.

### 2.2. Early Osmoregulatory Response Accompanied by hog1 and gpd1 Induction

**Figure 3:**
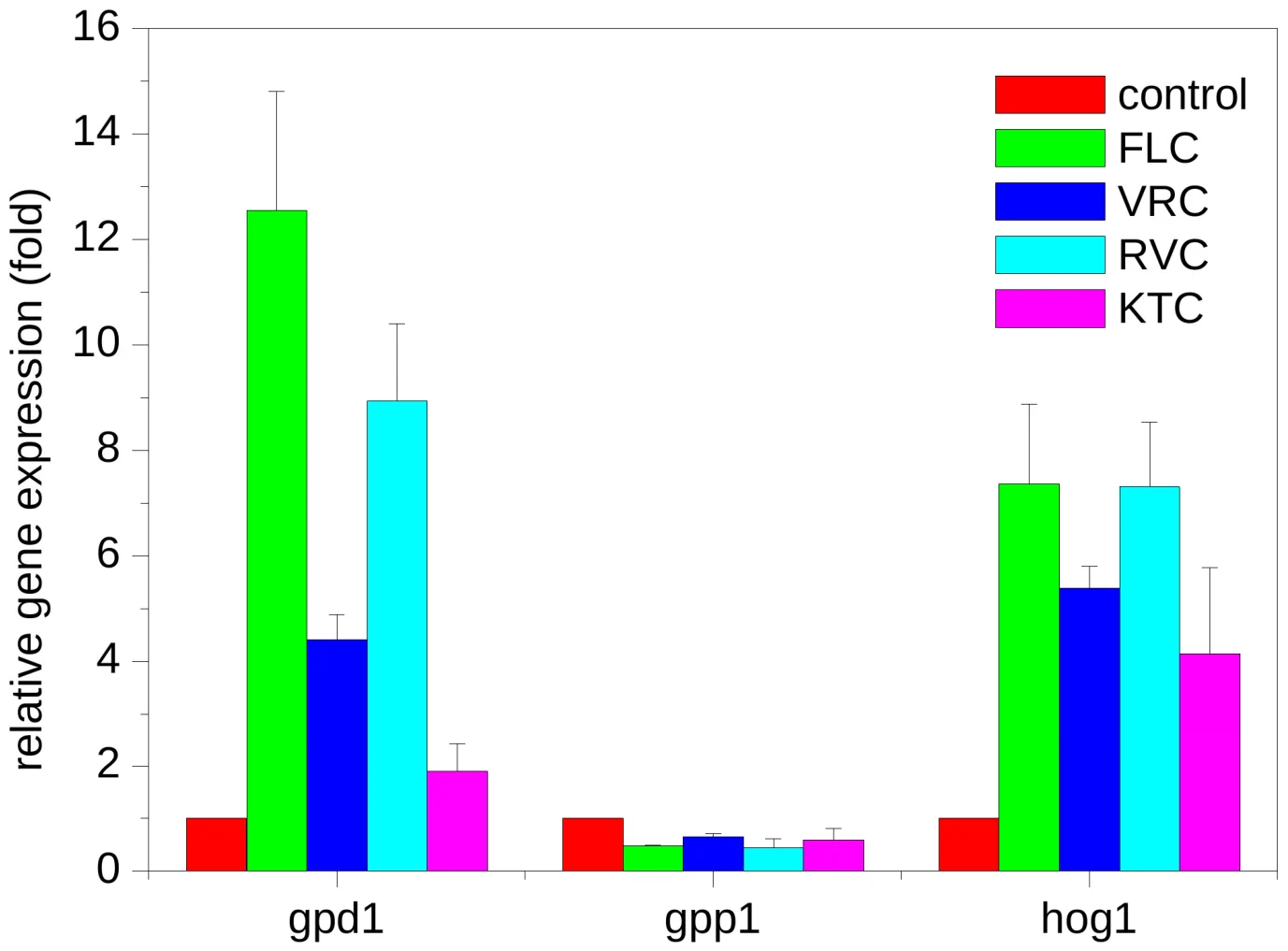
Relative expression of osmoregulatory genes in *Neurospora crassa* after 2 h of exposure to fluconazole (FLC), voriconazole (VRC), ravuconazole (RVC), or ketoconazole (KTC). The graph shows data from one representative biological experiment; error bars denote the standard deviation of three technical replicates within that experiment and do not represent between-experiment biological variability. Inferential analyses were performed on ΔCt values from three independent biological replicates using one-way ANOVA followed by Dunnett’s multiple-comparison test. Exact adjusted *p*-values are reported in Supplementary Table S3.

After 2 h, *hog1* expression differed significantly from the matched control after all four azoles, whereas *gpd1* differed after FLC, VRC, and RVC but not KTC. *gpp1* expression was significantly reduced after all four treatments (Figure 3; Supplementary Table S3).

The concordance between early glycerol accumulation and the treatment-dependent changes in *hog1* and *gpd1* is consistent with an early osmoregulatory response. Because HOG1 phosphorylation and pathway-specific mutants were not examined, these data do not demonstrate direct activation or functional involvement of the HOG pathway. The reduction in *gpp1* further shows that azole exposure did not uniformly induce all genes associated with glycerol metabolism.

### 2.3. Treatment-Dependent Feedback Regulation of Sterol and Membrane Homeostasis

**Figure 4:**
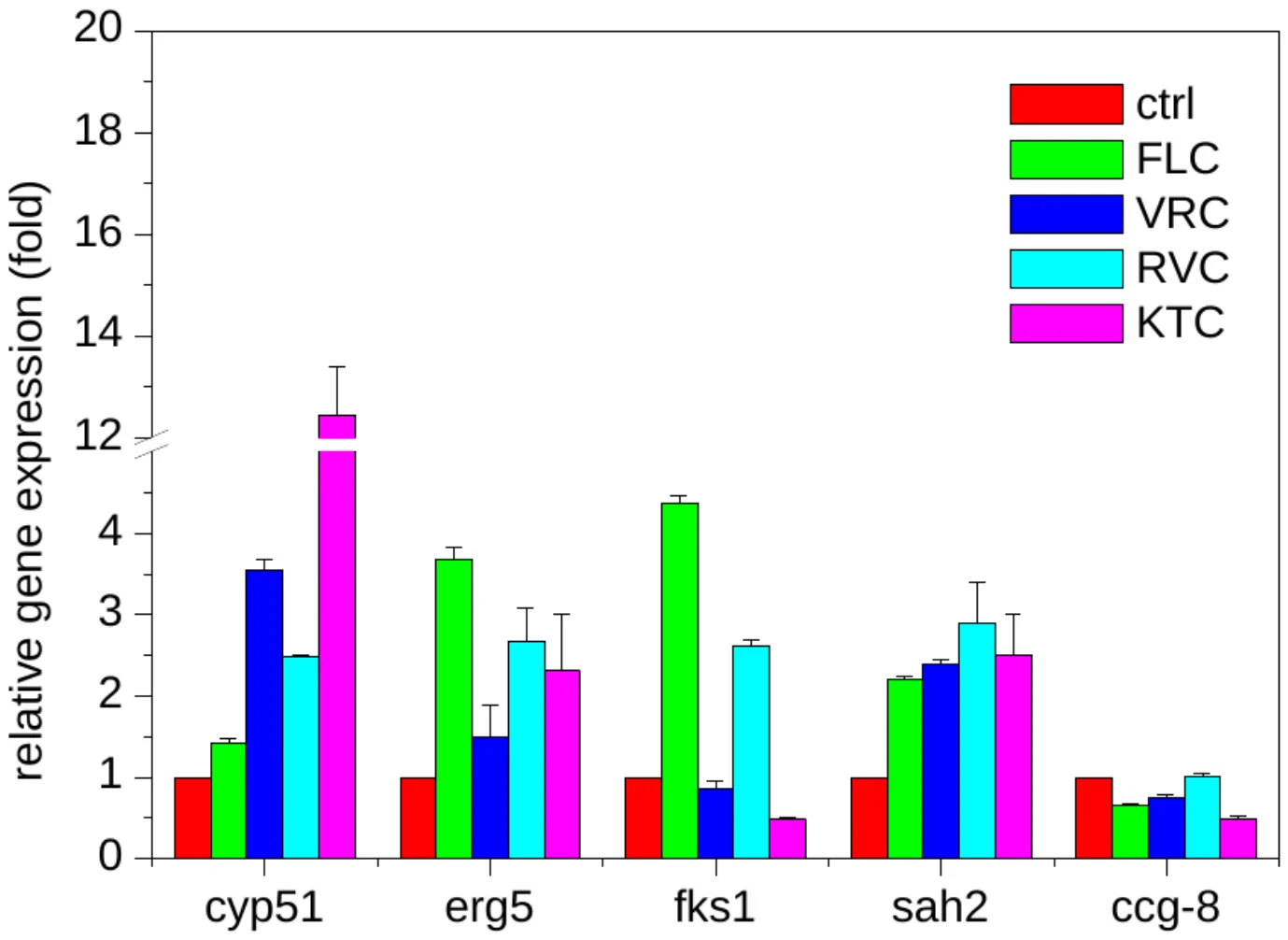
Relative expression of selected stress-response genes in *Neurospora crassa* after 2 h of azole exposure. Treatments are shown in the order control, fluconazole (FLC), voriconazole (VRC), ravuconazole (RVC), and ketoconazole (KTC). The graph shows data from one representative biological experiment; error bars denote the standard deviation of three technical replicates within that experiment and do not represent between-experiment biological variability. Inferential analyses were performed on ΔCt values from three independent biological replicates by one-way ANOVA followed by Dunnett’s multiple-comparison test. Exact adjusted *p*-values are reported in Supplementary Table S3.

After 2 h, the azole target gene *cyp51* differed significantly from the matched control under all four treatments. *erg5* differed after FLC and RVC, whereas VRC and KTC contrasts were not significant. The SREBP-like regulator *sah2* differed only after RVC exposure (Figure 4; Supplementary Table S3). These findings support rapid but treatment-selective feedback regulation of sterol homeostasis.

The β-1,3-glucan synthase gene *fks1* differed significantly from the matched control after all four treatments, with increases after FLC and RVC and lower expression after VRC and KTC. The broader stress-responsive transcription factor *ccg-8*, previously implicated in azole adaptation [13], differed after FLC, VRC, and KTC but not RVC. These concurrent transcriptional changes occurred within the same early exposure window, although their functional relationship was not examined.

### 2.4. Extensive Transcriptional Remodelling of Cell-Wall-Related Pathways

**Figure 5:**
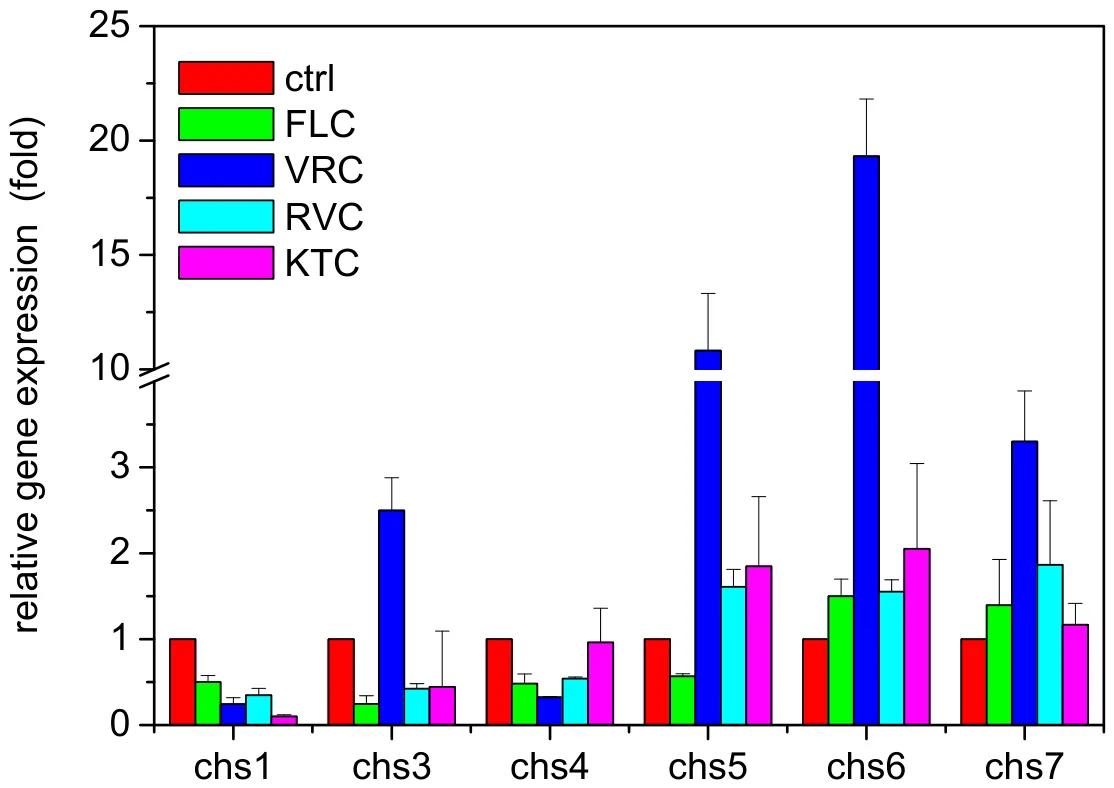
Relative expression of chitin synthase genes in *Neurospora crassa* after 2 h of exposure to fluconazole (FLC), voriconazole (VRC), ravuconazole (RVC), or ketoconazole (KTC). The graph shows data from one representative biological experiment; error bars denote the standard deviation of three technical replicates within that experiment and do not represent between-experiment biological variability. Inferential analyses were performed on ΔCt values from three independent biological replicates by one-way ANOVA followed by Dunnett’s multiple-comparison test, with exact adjusted *p*-values reported in Supplementary Table S3.

Chitin-synthase transcriptional responses after 2 h were gene- and treatment-dependent (Figure 5). *chs5* differed significantly under all four treatments; *chs4* differed after FLC, VRC, and RVC; and *chs7* differed after VRC and RVC. No treatment-versus-control contrast reached *p* < 0.05 for *chs3* or *chs6*. The data therefore support selective rather than uniform transcriptional remodelling of the chitin-synthase family.

**Figure 6:**
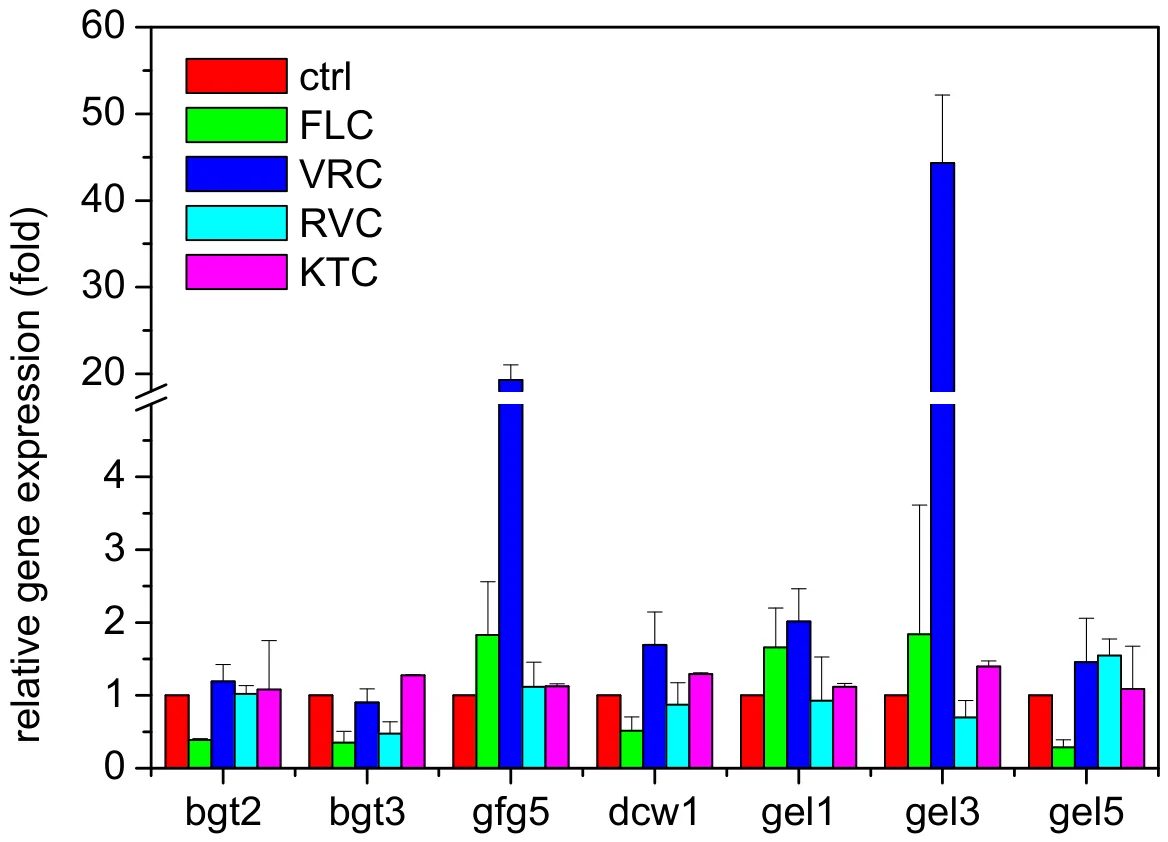
Relative expression of genes involved in cell-wall synthesis and remodelling in *Neurospora crassa* after 2 h of azole exposure. Treatments are fluconazole (FLC), voriconazole (VRC), ravuconazole (RVC), and ketoconazole (KTC). The graph shows data from one representative biological experiment; error bars denote the standard deviation of three technical replicates within that experiment and do not represent between-experiment biological variability. Inferential analyses were performed on ΔCt values from three independent biological replicates by one-way ANOVA followed by Dunnett’s multiple-comparison test. Exact adjusted *p*-values are reported in Supplementary Table S3.

To determine whether this response extended beyond chitin biosynthesis, we next examined genes involved in β-glucan cross-linking, glucan-chain elongation, and the incorporation of glycoproteins into the cell wall [14] (Figure 6).

VRC elicited the broadest set of significant changes, including *dfg5*, *gel1*, and *gel3*. FLC significantly affected *bgt2*, *bgt3*, *dfg5*, *dcw1*, and *gel5*; RVC also affected *bgt3*; no contrast reached *p* < 0.05 for KTC (Figure 6; Supplementary Table S3). Together with the chitin-synthase data, these findings indicate treatment-dependent transcriptional restructuring of selected cell-wall components rather than activation of a single compensatory biosynthetic pathway.

### 2.5. Treatment-Dependent Redox and Antioxidant Responses During Early Azole Exposure

**Figure 7:**
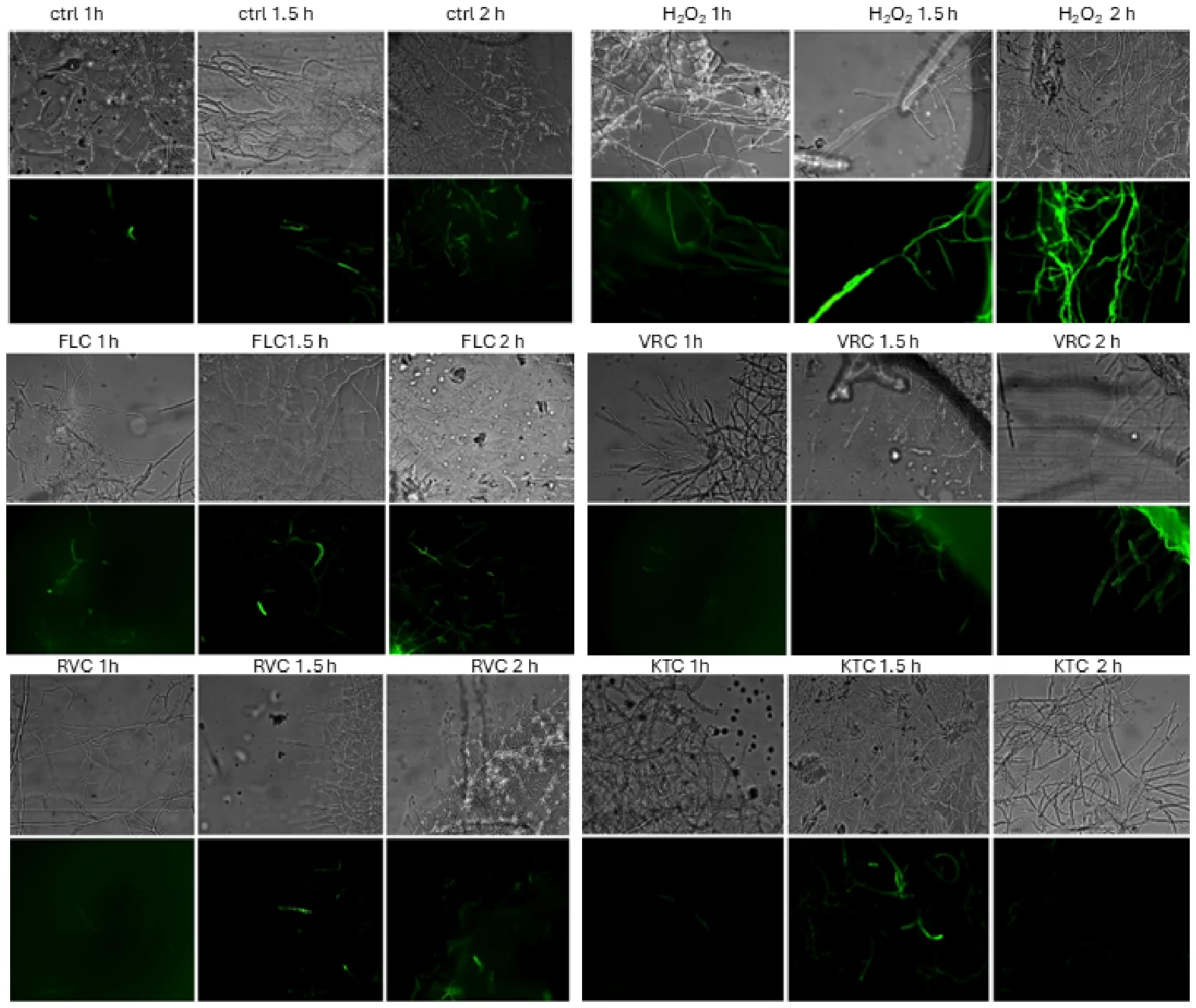
Qualitative detection of oxidant-associated fluorescence in *Neurospora crassa* after azole exposure. Mycelia were exposed to fluconazole (FLC), voriconazole (VRC), ravuconazole (RVC), or ketoconazole (KTC) for 1, 1.5, or 2 h. A matched DMSO vehicle control served as the negative control, and 25 mM H_2_O_2_ served as the positive control. H_2_DCF-DA fluorescence was visualised using a FITC filter set, and images were acquired at 200× total magnification. Representative transmitted-light and fluorescence images are shown. Fluorescence intensity was not quantified.

Oxidant-associated fluorescence was assessed by H_2_DCF-DA microscopy to determine whether detectable redox-associated changes accompanied the early azole response. Qualitative inspection showed low fluorescence in untreated controls and visually increased fluorescence in some azole-treated samples, most apparent after VRC exposure (Figure 7). Because fluorescence intensity was not quantified in the original analysis, these observations are interpreted qualitatively.

**Figure 8:**
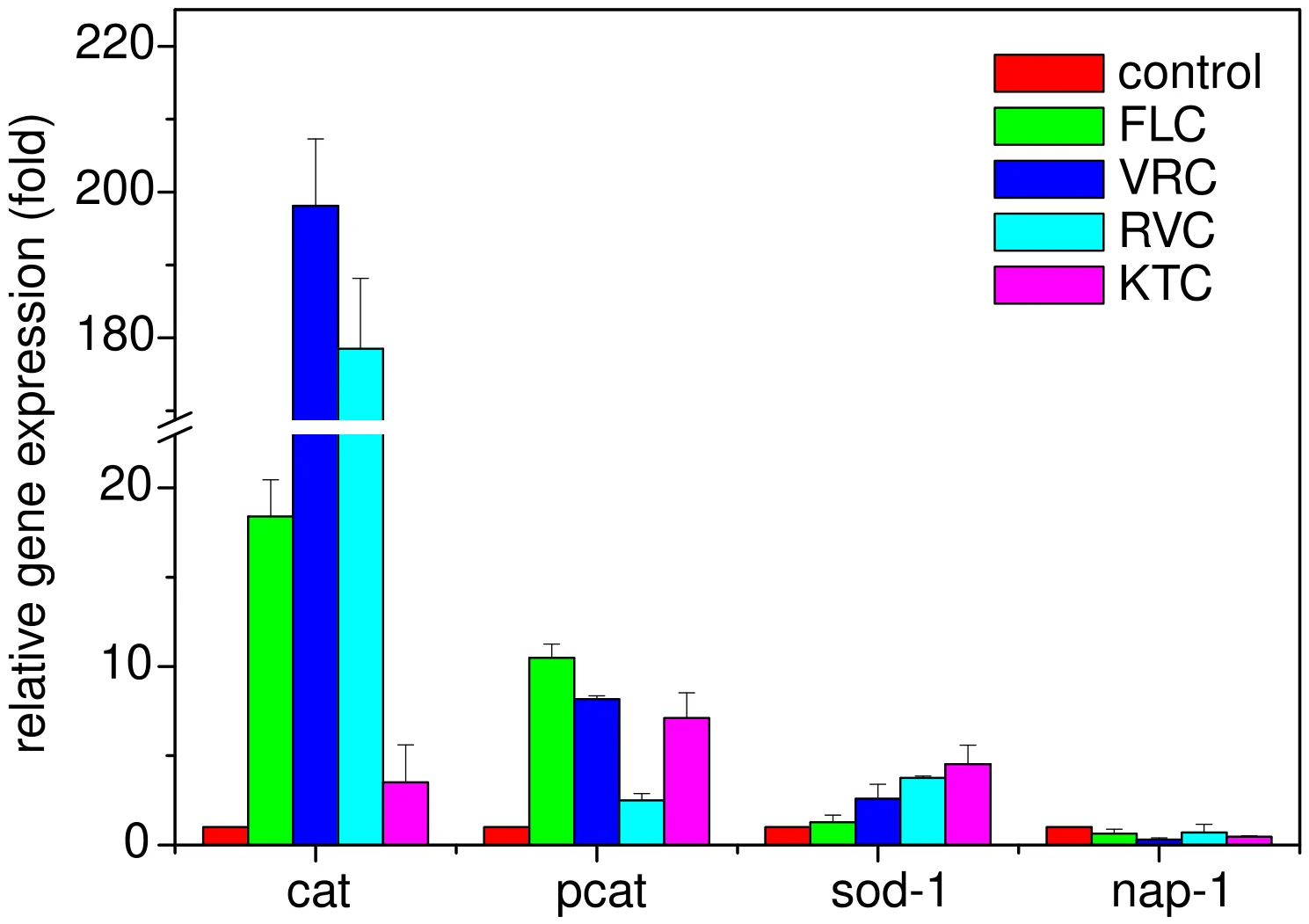
Relative expression of antioxidant-related genes in *Neurospora crassa* after 2 h of exposure to fluconazole (FLC), voriconazole (VRC), ravuconazole (RVC), or ketoconazole (KTC). The graph shows data from one representative biological experiment; error bars denote the standard deviation of three technical replicates within that experiment and do not represent between-experiment biological variability. Inferential analyses were performed on ΔCt values from three independent biological replicates by one-way ANOVA followed by Dunnett’s multiple-comparison test. Exact adjusted *p*-values are reported in Supplementary Table S3.

After 2 h, *cat*, *pcat*, and *sod-1* each differed significantly from their matched controls after all four azole treatments, whereas no treatment-versus-control contrast reached *p* < 0.05 for *nap-1* (Figure 8; Supplementary Table S3). NAP-1 is an AP-1-like bZIP transcription factor involved in oxidative-stress responses [15].

**Figure 9:**
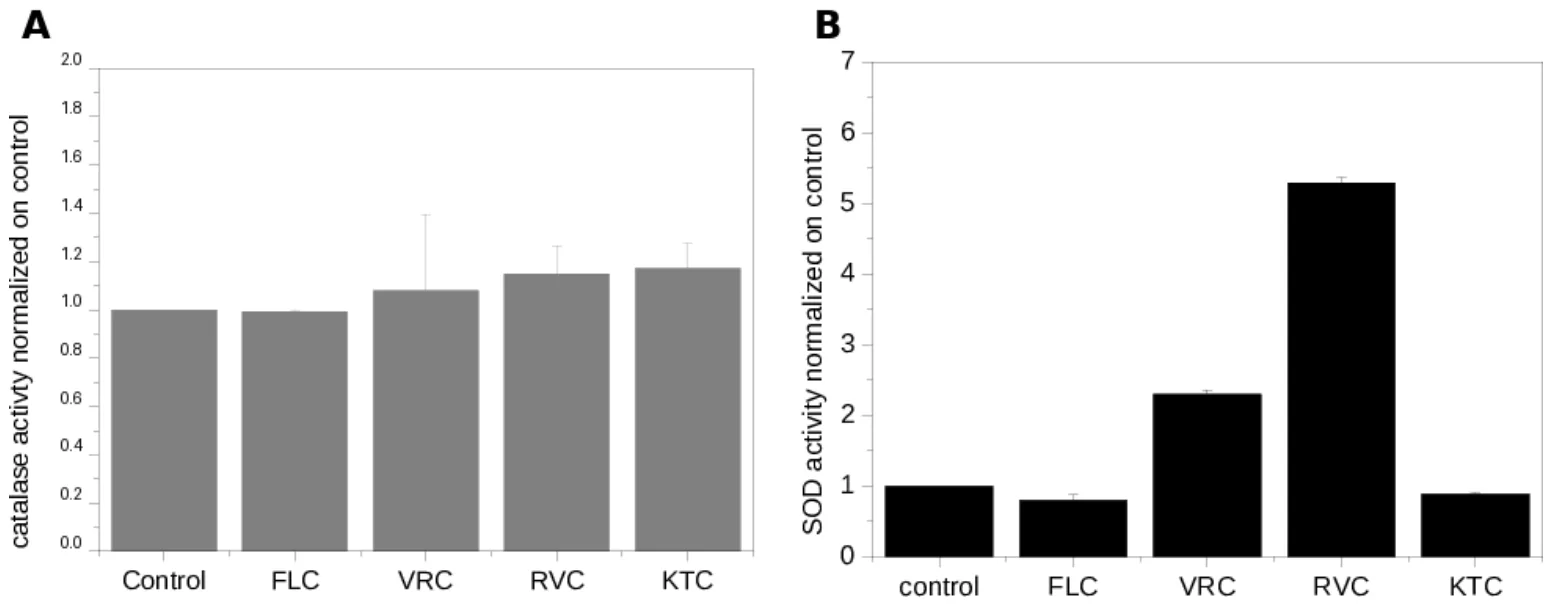
Catalase and superoxide dismutase activities in *Neurospora crassa* after 2 h of azole exposure. (**A**) Catalase and (**B**) SOD activities are expressed as fold changes relative to the corresponding matched vehicle control, which was normalised to 1. The graphs show representative experiments reflecting response patterns observed in independent experiments and are presented descriptively; no inferential statistical analysis was applied to the normalised fold-change values, and no significance indicators are shown.

Catalase and superoxide dismutase (SOD) activities were examined after 2 h of azole exposure (Figure 9). Catalase activity remained close to the matched vehicle-control level. Representative SOD-activity experiments showed larger fold changes after VRC and RVC than after FLC or KTC. The strong *cat* transcriptional response was not accompanied by a detectable increase in catalase activity. The SOD activity pattern likewise did not closely parallel *sod-1* transcript abundance at 2 h, indicating that transcript levels were not directly predictive of enzymatic activity under the conditions examined.

### 2.6. Qualitative Changes in Calcofluor White Staining Patterns

**Figure 10:**
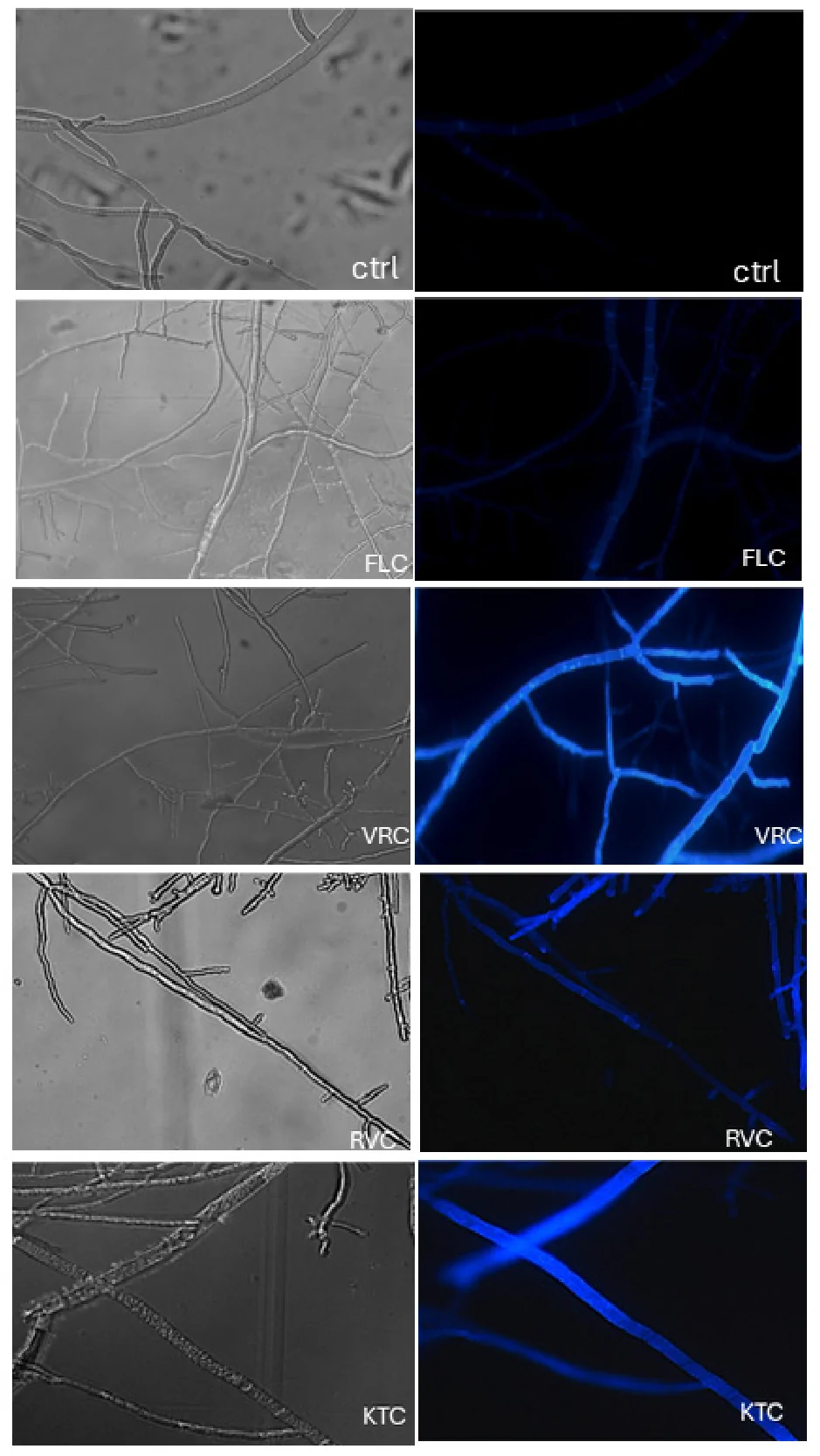
Qualitative Calcofluor White staining of *Neurospora crassa* after 2 h of azole exposure. Representative transmitted-light and fluorescence micrographs are shown in the order control (ctrl), fluconazole (FLC), voriconazole (VRC), ravuconazole (RVC), and ketoconazole (KTC). Images were acquired at 200× total magnification. In the paired panels, transmitted-light images are shown in grayscale and Calcofluor White-associated fluorescence is shown in blue. These images are descriptive and do not quantify chitin abundance.

Because azole-induced membrane stress may require compensatory adjustment of the cell wall [16], Calcofluor White staining was used to examine cell-wall-associated fluorescence after 2 h of exposure. Untreated hyphae displayed comparatively weak and uniform staining, whereas treated samples showed heterogeneous staining patterns in the examined fields (Figure 10). These results are presented as descriptive morphological findings.

**Figure 11:**
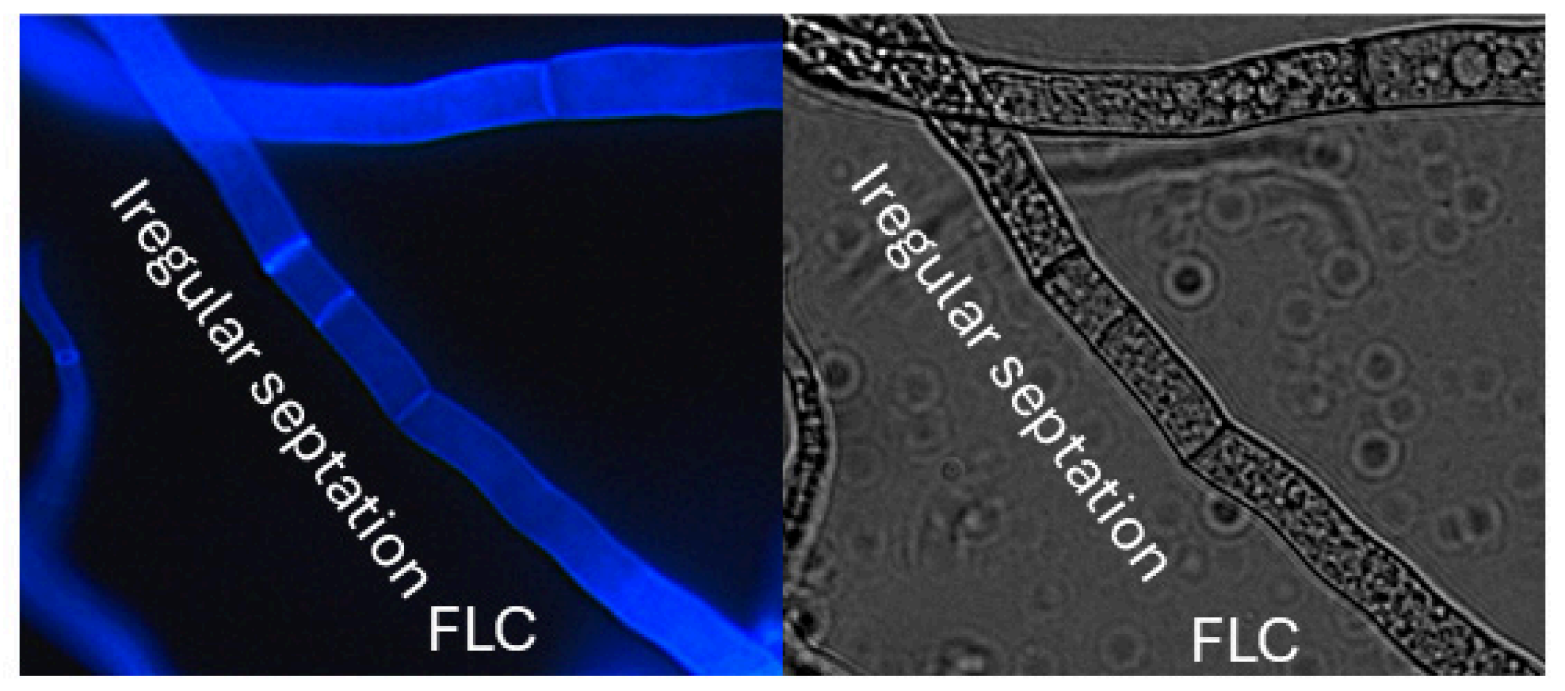
Qualitative observation of irregular septation in *Neurospora crassa* hyphae after 2 h of fluconazole (FLC) exposure. Chitin-associated staining was visualised with Calcofluor White at 400× total magnification. The transmitted-light image is shown in grayscale and Calcofluor White-associated fluorescence is shown in blue.

**Figure 12:**
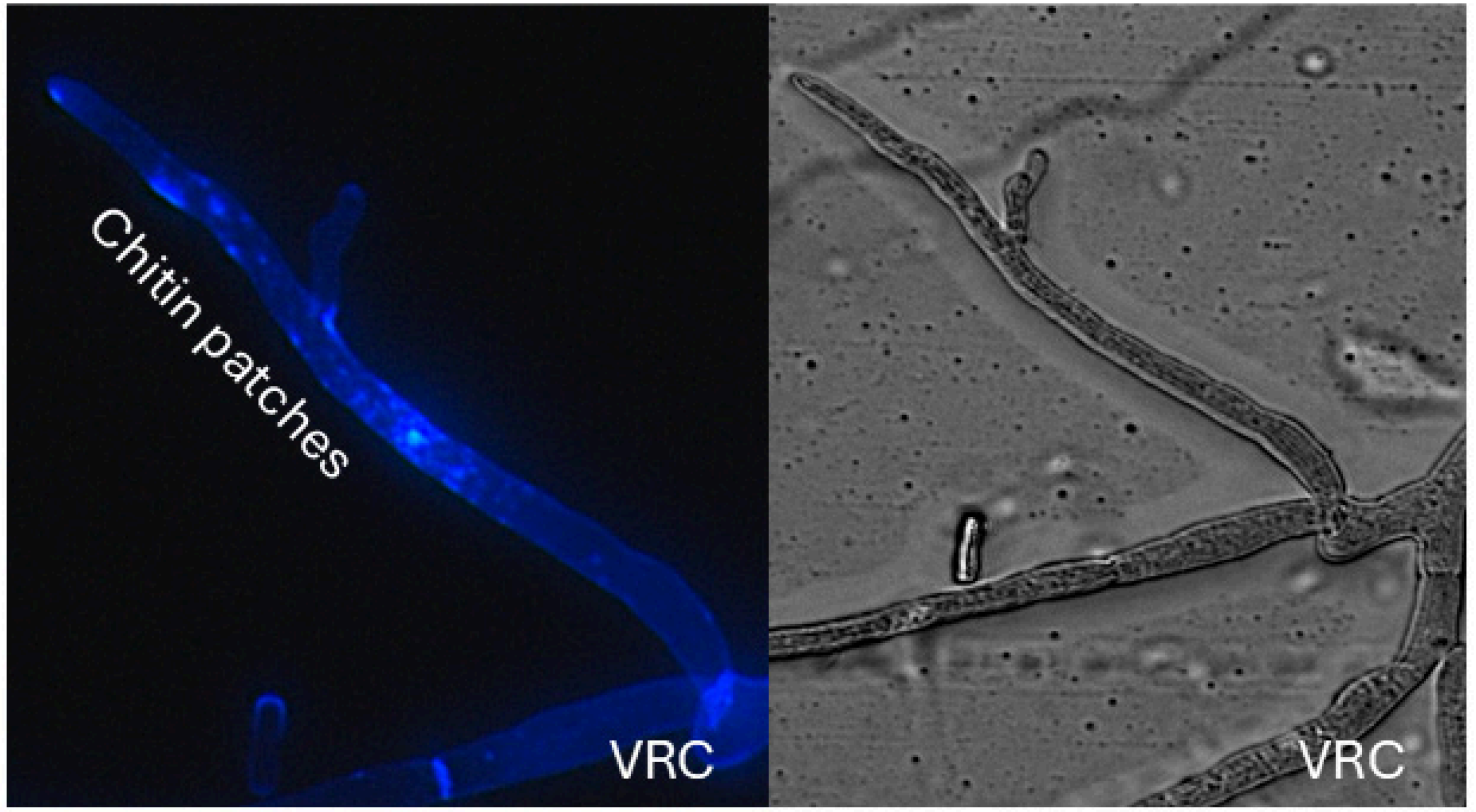
Qualitative observation of localised regions of enhanced Calcofluor White staining in *Neurospora crassa* hyphae after 2 h of voriconazole (VRC) exposure. Images were acquired at 400× total magnification. The transmitted-light image is shown in grayscale and Calcofluor White-associated fluorescence is shown in blue.

Representative higher-magnification images showed irregularly positioned septa after FLC treatment (Figure 11) and discrete regions of locally enhanced Calcofluor White staining after VRC exposure (Figure 12). The VRC-associated staining pattern occurred under a condition in which *chs7* expression differed significantly from the matched control. However, this co-occurrence does not establish a causal relationship between gene expression and morphology.

## 3. Discussion

The present study shows that early azole exposure in *Neurospora crassa* is accompanied by changes in several cellular processes rather than by a single dominant response. Changes in colony growth occurred alongside osmolyte accumulation, sterol-pathway feedback, antioxidant responses, and transcriptional reprogramming of genes involved in cell-wall biosynthesis and remodelling. These observations indicate that multiple homeostatic processes respond within the same early phase of azole exposure. However, the present study was not designed to establish direct functional relationships among these processes.

The present findings complement our previous study [11]. The earlier work characterised a later phase of the response to azole exposure, with particular emphasis on xenobiotic detoxification, including CYP65/ABCC-associated mechanisms, and increased chitin synthesis. In contrast, the present study focuses on the first 1–6 h after azole exposure and examines physiological and transcriptional events not addressed previously, including glycerol accumulation, osmoregulatory gene expression, early sterol-pathway feedback, antioxidant responses, and a broader panel of cell-wall-remodelling genes. The omission of CYP65 and ABCC genes from the present analysis was intentional because these pathways had already been characterised in the previous study. The early cell-wall transcriptional and morphological changes observed here may be related to the more pronounced chitin-associated phenotype reported at the later exposure stage [11]; however, the two studies used independent experimental datasets and do not establish a direct temporal sequence. Together with our previous findings, the present results extend the characterisation of the azole response by focusing on its early phase.

Osmotic adjustment was among the earliest responses observed after azole exposure. Inhibition of ergosterol biosynthesis is expected to alter membrane fluidity, permeability, and ion homeostasis, thereby creating an osmoregulatory challenge [17,18]. The rapid increase in intracellular glycerol, together with induction of *hog1* and *gpd1*, is consistent with an early osmoregulatory response and with possible involvement of HOG-related signalling. However, because HOG1 phosphorylation and pathway-specific mutants were not examined, direct activation or functional involvement of the HOG pathway cannot be concluded from the present data. The distinct glycerol trajectories observed with the individual treatments further indicate that the early osmotic response is dynamic and treatment-dependent.

HOG-related signalling is known to interact with fungal redox homeostasis, and activated Hog1 can regulate genes involved in antioxidant defence [19]. In the present study, *hog1* induction occurred within the same early exposure window as changes in catalase- and SOD-encoding transcripts, whereas *nap-1*, encoding an AP-1-like oxidative-stress transcription factor [15], remained comparatively stable. Together with the limited oxidant-associated fluorescence observed qualitatively, these findings are compatible with an early redox response. However, because HOG1 activation was not measured and the H_2_DCF-DA microscopy was qualitative [20], the present data do not establish a mechanistic link between HOG-related signalling and the antioxidant response.

The antioxidant-response data indicate that transcript abundance and enzyme activity were not tightly coupled at 2 h. Although *cat* transcript abundance increased, catalase activity remained close to control levels. Representative SOD-activity experiments showed the largest fold change after RVC and a smaller increase after VRC, whereas the corresponding *sod-1* transcriptional changes were more modest. These discrepancies between gene expression and enzyme activity may reflect response kinetics, translational or post-translational regulation, enzyme maturation or stability, or pre-existing enzyme pools; for catalases, diversity in regulation and cellular roles is well documented [21,22].

In parallel with osmotic and antioxidant responses, *cyp51* differed significantly after all four azoles, whereas *erg5* differed after FLC and RVC and the sterol-responsive regulator *sah2* differed only after RVC. These results support rapid but treatment-selective sterol-homeostasis feedback [7,8,23,24]. The pattern remained narrower than the broader transcriptional response reported after prolonged azole exposure [11], supporting a temporal distinction between early selective feedback and the later response.

The distinct patterns of *cyp51*, *erg5*, *sah2*, *fks1*, and *chs7* expression, together with the differential SOD response, show that the four azole treatments did not produce identical early-response profiles. Differences in drug affinity, intracellular accumulation, membrane partitioning, sterol intermediates, or secondary cellular effects may contribute to these patterns. However, the MIC_80_ concentrations used here were originally determined with conidia; therefore, differences in the effective stress imposed on pre-grown mycelia may also contribute. The observed treatment-dependent responses should consequently not be attributed solely to intrinsic differences among the azole compounds.

Cell-wall-related transcriptional changes were extensive but treatment-dependent. Changes in plasma-membrane organisation and tension can interact with pathways maintaining cell-wall integrity [25], and the fungal cell wall is continuously remodelled during growth and environmental stress [26,27,28]. VRC produced the broadest set of significant responses, including *chs4*, *chs5*, *chs7*, *dfg5*, *gel1*, and *gel3*. Other azoles elicited narrower patterns. Calcofluor White microscopy provided qualitative evidence of altered hyphal organisation and localised changes in cell-wall-associated staining.

Taken together, the results show that short-term inhibition of sterol biosynthesis is accompanied by multiple early cellular responses, including altered colony growth, glycerol accumulation, changes in osmoregulatory gene expression, antioxidant responses, sterol-pathway feedback, and transcriptional remodelling of cell-wall-related pathways. These processes occurred within overlapping time windows but were assessed using independent assays and were not functionally linked in the present study. The observed patterns should therefore be interpreted as concurrent components of the early azole-stress response rather than as evidence of a single integrated signalling programme.

Several limitations should be considered when interpreting these findings. HOG-pathway involvement was inferred from glycerol accumulation and gene-expression patterns without direct measurement of HOG1 phosphorylation or analysis of pathway mutants. H_2_DCF-DA and Calcofluor White microscopy were qualitative; neither fluorescence intensity nor morphometric endpoints were quantified prospectively. H_2_DCF-DA does not selectively report a single reactive oxygen species [20], and Calcofluor White staining is not a direct quantitative measurement of chitin abundance. For the Calcofluor White series, the available records did not support a reliable retrospective count of independent biological experiments. Reference-gene concordance was assessed in a representative biological replicate rather than by a formal multi-replicate stability analysis. In addition, conidium-derived MIC_80_ concentrations cannot be assumed to impose identical effective stress intensities on 24-h pre-grown mycelia. Finally, *Neurospora crassa* is a model filamentous fungus, and the conservation and functional significance of these responses in clinically relevant species remain to be established [29].

The present study therefore provides a working framework for the early response of *Neurospora crassa* to azole stress, in which short-term exposure is accompanied by changes in sterol homeostasis, osmotic adjustment, antioxidant responses, and cell-wall-related pathways. Future studies combining pathway-specific mutants, biochemical analyses, quantitative microscopy, recovery assays, susceptibility measurements, and higher temporal resolution will be required to determine how these processes interact and whether they contribute to functional adaptation or tolerance.

## 4. Materials and Methods

### 4.1. Strain and Cultivation Conditions

The wild-type strain *Neurospora crassa* FGSC 2489 (mating type A) was obtained from the Fungal Genetics Stock Center (FGSC, Manhattan, KS, USA). Conidial cultures were prepared on Vogel’s minimal medium [30] supplemented with 1.5% (*w*/*v*) sucrose and solidified with 1.5% (*w*/*v*) agar. Cultures were maintained at 30 °C in the dark for 2 days and then for up to 7 days under circadian conditions.

For azole exposure experiments, sterile cellophane overlays were used to facilitate the transfer of pre-grown mycelia in the exponential growth phase. Colonies were pre-cultivated on cellophane-covered agar plates at 30 °C for 24 h in the dark before transfer to media containing antifungal compounds.

### 4.2. Antifungal Compounds and Exposure Conditions

Fluconazole (FLC), voriconazole (VRC), and ketoconazole (KTC) were obtained from Sigma-Aldrich (St. Louis, MO, USA). Ravuconazole (RVC) was synthesised at the Institute of Organic Chemistry, Catalysis and Petrochemistry, Slovak University of Technology in Bratislava, for commercial purposes. The supplied compound was analytical grade and optically pure, and its chemical identity was confirmed by NMR spectroscopy (Varian, Inc., Palo Alto, CA, USA).

Stock solutions were prepared in dimethyl sulfoxide (DMSO) and diluted to the previously determined MIC_80_ concentrations in the order FLC, 15 µg/mL; VRC, 0.3 µg/mL; RVC, 0.6 µg/mL; and KTC, 0.4 µg/mL [11]. These concentrations were determined for conidia of the same *Neurospora crassa* strain and were used as standardised reference concentrations for short-term exposure of pre-grown mycelia; they were not assumed to impose identical stress intensities on 24-h mycelia. In every experimental series, including growth, glycerol, RT-qPCR, antioxidant-enzyme, H_2_DCF-DA, and Calcofluor White assays, the corresponding matched control received the same amount of DMSO as the azole-treated sample. Final DMSO did not exceed 1% (*v*/*v*).

### 4.3. Preparation of Pre-Cultures and Growth Monitoring

Pre-cultures were prepared on Vogel’s minimal medium supplemented with 1.5% (*w*/*v*) sucrose and solidified with 1.5% (*w*/*v*) agar in 90-mm Petri dishes. The medium surface was covered with sterile cellophane and inoculated with a conidial suspension.

Conidia were harvested from 7-day-old cultures using sterile water and adjusted to a final concentration of 2.5 × 10^5^ conidia/mL. A 5-µL aliquot of the suspension was applied to sterile paper discs placed on cellophane-covered agar and incubated for 24 h at 30 °C in the dark.

After incubation, colonies were transferred together with the supporting cellophane membrane onto agar containing the MIC_80_ concentration of each azole or the corresponding matched vehicle control. Two perpendicular colony diameters were measured manually with a ruler and averaged. The initial diameter was recorded immediately before transfer (D_0_), and diameters were recorded at each subsequent time point (D_t_). Net post-transfer growth was calculated as D_t_ − D_0_ and expressed relative to the corresponding increment of the matched vehicle control.

### 4.4. Quantification of Intracellular Glycerol

Intracellular glycerol levels were measured after 2, 4, and 6 h of exposure to azole antifungals at MIC_80_ concentrations. Mycelial samples were harvested from cellophane overlays, frozen in liquid nitrogen, homogenised, and weighed.

Each frozen, homogenised mycelial sample was resuspended in distilled water, vortexed, and incubated at 90 °C for 15 min. After centrifugation at 13,000× *g* for 2 min, the supernatant was analysed using a colourimetric Glycerol Assay Kit (Sigma-Aldrich, St. Louis, MO, USA) according to the manufacturer’s instructions. Absorbance was measured at 570 nm. Glycerol content calculated from the calibration curve was normalised to the mass of frozen mycelial powder used for the assay and expressed as µg glycerol per mg frozen mycelial powder. Three independent biological replicates were analysed.

### 4.5. Detection of Intracellular Oxidant-Associated Fluorescence

Pre-cultivated colonies were transferred to agar containing an azole, the corresponding matched DMSO vehicle control, or 25 mM hydrogen peroxide as a positive control. Final DMSO did not exceed 1% (*v*/*v*).

After 1, 1.5, or 2 h of exposure, 20 µM H_2_DCF-DA was applied directly to the mycelial surface, followed by incubation at 30 °C for 30 min in the dark. Samples were washed with 0.89% NaCl, mounted, and examined using an Axio Imager A1 fluorescence microscope with an Axiocam ICC 1 camera and AxioVision 4.8 software (Carl Zeiss Microscopy GmbH, Jena, Germany). Fluorescence was visualised using a FITC filter set (excitation/emission 495/519 nm). All samples were examined under identical imaging conditions, including fluorescence excitation intensity, acquisition settings, and magnification (20× objective × 10× eyepiece × 1× additional magnification), allowing direct qualitative comparison of fluorescence patterns between control and treated samples. Five fields per biological sample were scanned, and one representative field was selected for evaluation. Three independent biological experiments were performed.

### 4.6. Determination of Catalase and Superoxide Dismutase Activities

Catalase activity was measured using the Catalase Assay Kit MAK531 (Merck KGaA, Darmstadt, Germany) according to the manufacturer’s instructions. SOD activity was determined using the SOD Determination Kit (Merck KGaA, Darmstadt, Germany), based on the WST-1 [2-(4-iodophenyl)-3-(4-nitrophenyl)-5-(2,4-disulfophenyl)-2H-tetrazolium] colourimetric assay. Pre-cultivated *Neurospora crassa* mycelia were transferred to azole-containing agar plates. After 2 h of exposure, the mycelia were rapidly frozen in liquid nitrogen and ground to a fine powder. Approximately 20 mg of frozen mycelium was homogenised in 100 µL of cold phosphate-buffered saline by vortexing. Homogenates were centrifuged at 14,000× *g* for 10 min at 4 °C, and the clear supernatants were collected. Total protein concentration was determined using the Bradford assay [31].

For the catalase assay, 10 µL of diluted supernatant was added to 90 µL of freshly prepared 50 µM H_2_O_2_ substrate and incubated for 30 min at room temperature. Detection reagent was then added, residual H_2_O_2_ was quantified at 570 nm, and catalase activity was calculated from an H_2_O_2_ standard curve and normalised to total protein. For the SOD assay, sample extract was mixed with WST and xanthine-oxidase working solutions, incubated at 37 °C for 20 min, and read at 450 nm. SOD activity was calculated from the standard curve and was normalised to total protein in the sample. Sample-specific blank wells corrected background absorbance and colour. Direct azole-specific chemical interference with the WST-1 reaction was not tested separately. Relative catalase and SOD activities were expressed as fold changes relative to the corresponding matched vehicle control, normalised to 1. Assays were performed in three independent experiments.

### 4.7. Chitin Staining and Fluorescence Microscopy

Chitin distribution was visualised using Calcofluor White (CFW; Sigma-Aldrich, St. Louis, MO, USA). *Neurospora crassa* mycelia were pre-cultivated for 24 h on Vogel’s medium with sterile cellophane overlays, transferred to azole-containing or control medium, and incubated for 2 h.

After incubation, mycelia were stained with Calcofluor White diluted 1:20 in sterile water for 15 min at 30 °C in the dark and washed three times with 0.89% NaCl. Mycelial fragments were mounted in saline and examined using an Axio Imager A1 fluorescence microscope equipped with an Axiocam ICC 1 camera and AxioVision 4.8 software (Carl Zeiss Microscopy GmbH, Jena, Germany). Fluorescence was detected using a CFW/DAPI-compatible filter set (Carl Zeiss Microscopy GmbH, Jena, Germany). The images were evaluated qualitatively. All samples were examined under identical imaging conditions, including fluorescence excitation intensity, acquisition settings, and magnification (20× objective × 10× eyepiece × 1× additional magnification), allowing direct qualitative comparison of fluorescence patterns between control and treated samples. Five fields per biological sample were scanned, and one representative field was selected for evaluation.

### 4.8. RNA Isolation and Quantitative PCR

Total RNA was isolated from mycelial samples homogenised in liquid nitrogen using TRI Reagent (Sigma-Aldrich, St. Louis, MO, USA), as described previously [11]. Isolated RNA was treated with DNase I to remove genomic DNA contamination and reverse-transcribed using AllScript Reverse Transcriptase (Biotechrabbit, Berlin, Germany) with oligo(dT)_16_ primers.

RT-qPCR was performed using CAPITAL qPCR Green Master Mix (Biotechrabbit, Berlin, Germany) on aqTower3 Real-Time PCR system (Analytik Jena AG, Jena, Germany). Primer sequences used for RT-qPCR are listed in Supplementary Table S2. Relative expression was calculated by the 2^−ΔΔCt^ method [32] with histone H3 (hH3; NCU01635) as the reference gene. The importance of reference-gene validation in RT-qPCR is well established [33,34]. During initial method validation, selected target-gene Ct values from a representative biological replicate were independently normalised to β-tubulin and hH3; the overall treatment-response patterns were qualitatively concordant, and hH3 was subsequently used consistently for the complete dataset (Supplementary Table S4). Three independent biological replicates were used for inferential analyses on ΔCt values. Genes and NCU identifiers are listed in Supplementary Table S1.

### 4.9. Statistical Analysis

For RT-qPCR analyses, statistical evaluation was performed using ΔCt values obtained from three independent biological replicates. For comparisons of multiple azole treatments with a common 1% DMSO control, differences were analysed by one-way analysis of variance (ANOVA), followed by Dunnett’s multiple-comparison test, with the corresponding vehicle control as the reference group. Where ketoconazole was analysed in an independent experimental set for the same gene sets, it was compared with its corresponding matched control. Exact adjusted *p*-values are provided in Supplementary Table S3. For the glycerol time-course experiment, the effects of treatment and exposure time were analysed by two-way ANOVA, with treatment and time as independent factors, including their interaction. Dunnett’s multiple-comparison test was subsequently used to compare individual azole treatments with the corresponding vehicle control at each time point. Detailed results, including adjusted *p*-values, are provided in Supplementary Table S5. Data shown in representative RT-qPCR experiments are presented as relative gene expression, with error bars representing the standard deviation (SD) of technical replicates within the respective experiment. Catalase and SOD activities are presented descriptively as fold changes relative to the corresponding vehicle control, which was normalised to 1. These normalised representative data were not subjected to inferential statistical testing. Similarly, H_2_DCF-DA and Calcofluor White microscopy experiments were evaluated qualitatively and were not subjected to quantitative statistical analysis. Statistical differences were considered significant at *p* < 0.05.

## 5. Conclusions

Exposure of *Neurospora crassa* to azole antifungals elicited rapid physiological and transcriptional responses characterised by changes in relative colony growth, glycerol accumulation and osmotic adjustment, transcriptional changes in osmoregulatory genes, treatment-dependent antioxidant responses, selective feedback regulation of sterol-homeostasis genes, extensive transcriptional remodelling of cell-wall-related pathways, and altered cell-wall-associated staining patterns.

Overall, the findings show that several homeostatic processes respond during the first hours of azole stress. Their temporal co-occurrence provides a basis for future mechanistic studies, but the present data do not establish direct functional integration among these responses or demonstrate that they confer recovery, tolerance, or longer-term adaptation. Whether any of these early stress-responsive processes can be therapeutically exploited will require direct testing in pathogenic fungi and experimental evaluation using combination-treatment approaches.

## Data Availability

The data presented in this study are available within the article and its Supplementary Materials. Additional data are available from the corresponding author upon reasonable request.

## Supplementary Materials

The following supporting information can be downloaded at: https://www.mdpi.com/article/10.3390/antibiotics15080811/s1, Table S1, genes analysed by RT-qPCR, including NCU identifiers, encoded proteins, and primary biological roles; Table S2, primers used for RT-qPCR; Table S3, one-way ANOVA and Dunnett-adjusted multiple-comparison results for RT-qPCR ΔCt values; Table S4, representative β-tubulin versus histone H3 normalisation comparison; and Table S5, two-way ANOVA and Dunnett-adjusted comparisons for glycerol accumulation.
