# Supplementary material for: Early Cellular Responses of Neurospora crassa to Azole Stress: Osmotic Adjustment, Redox Buffering, Sterol-Pathway Feedback, and Cell Wall Remodelling
Source: Antibiotics (Basel). 2026 Aug 19;15(8):811. doi: 10.3390/antibiotics15080811 (PMC13509159; doi:10.3390/antibiotics15080811)
Supplement: Supplementary file 1 [file antibiotics-15-00811-s001.zip › antibiotics-4494227-supplementary.pdf]

Supplementary Materials

Early Cellular Responses of *Neurospora crassa* to Azole Stress: Osmotic Adjustment, Redox Buffering, Sterol-Pathway Feedback, and Cell Wall Remodelling

Tomáš Pagáč[1],*, Ján Víglaš[1] and Petra Olejníková[1],*

[1] Institute of Biochemistry and Microbiology, Faculty of Food and Chemical Technology, Slovak University of Technology in Bratislava, Radlinského 9, 812 37 Bratislava, Slovakia

** Correspondence: tomaspagac1@gmail.com; petra.olejnikova@stuba.sk*

Supplementary Table S1. Genes analysed in this study

Genes analysed by RT-qPCR in *Neurospora crassa* following azole exposure, grouped according to their primary biological function.

Functional module	Gene	NCU ID	Encoded protein	Primary biological role
Ergosterol biosynthesis and sterol regulation	*cyp51*	NCU02624	Cytochrome P450 51 (lanosterol 14α-demethylase)	Ergosterol biosynthesis
Ergosterol biosynthesis and sterol regulation	*erg3*	NCU06207	C-5 sterol desaturase	Ergosterol biosynthesis
Ergosterol biosynthesis and sterol regulation	*erg4*	NCU01333	Δ(24(24¹))-sterol reductase	Ergosterol biosynthesis
Ergosterol biosynthesis and sterol regulation	*erg5*	NCU05278	Cytochrome P450 61 (C-22 sterol desaturase)	Ergosterol biosynthesis
Ergosterol biosynthesis and sterol regulation	*erg6*	NCU03006	Sterol C-24 methyltransferase	Ergosterol biosynthesis
Ergosterol biosynthesis and sterol regulation	*erg24*	NCU08762	C-14 sterol reductase	Ergosterol biosynthesis
Ergosterol biosynthesis and sterol regulation	*sah2*	NCU04731	SREBP-like transcription factor SAH-2	Regulation of sterol homeostasis
HOG signalling and osmoadaptation	*os-2 (hog1 homologue)*	NCU07024	Mitogen-activated protein kinase OS-2	Osmotic stress signalling
HOG signalling and osmoadaptation	*gpd1*	NCU00742	Glycerol-3-phosphate dehydrogenase	Glycerol biosynthesis
HOG signalling and osmoadaptation	*gpp1 (pdx-3)*	NCU03068	Glycerol-3-phosphate phosphatase	Glycerol biosynthesis
Oxidative stress response	*nap-1*	NCU03905	AP-1-like bZIP transcription factor	Regulation of oxidative stress response
Oxidative stress response	*cat-1 (cat)*	NCU08791	Catalase-1	Hydrogen peroxide detoxification
Oxidative stress response	*cat-2 (pcat)*	NCU05770	Catalase-peroxidase	Hydrogen peroxide detoxification
Oxidative stress response	*sod-1*	NCU02133	Cu/Zn superoxide dismutase	Superoxide detoxification
Cell wall synthesis and remodelling	*fks1*	NCU06871	β-1,3-glucan synthase	β-1,3-glucan biosynthesis
Cell wall synthesis and remodelling	*ccg-8*	NCU09686	Clock-controlled gene-8 (CCG-8 transcription factor)	Stress-responsive transcription factor
Cell wall synthesis and remodelling	*chs1*	NCU03611	Chitin synthase I	Chitin biosynthesis
Cell wall synthesis and remodelling	*chs3*	NCU04251	Chitin synthase III	Chitin biosynthesis
Cell wall synthesis and remodelling	*chs4*	NCU09324	Chitin synthase IV	Chitin biosynthesis
Cell wall synthesis and remodelling	*chs5*	NCU04352	Chitin synthase V	Chitin biosynthesis
Cell wall synthesis and remodelling	*chs6*	NCU05268	Chitin synthase VI	Chitin biosynthesis
Cell wall synthesis and remodelling	*chs7*	NCU04350	Chitin synthase VII	Chitin biosynthesis
Cell wall synthesis and remodelling	*bgt2*	NCU09175	GPI-anchored β-1,3-endoglucanase	β-glucan remodelling
Cell wall synthesis and remodelling	*bgt3*	NCU09326	Putative β-glucan remodelling protein	Cell wall remodelling
Cell wall synthesis and remodelling	*gel1*	NCU08909	β-1,3-glucanosyltransferase GEL1	β-glucan remodelling
Cell wall synthesis and remodelling	*gel3*	NCU01162	β-1,3-glucanosyltransferase GEL3	β-glucan remodelling
Cell wall synthesis and remodelling	*gel5*	NCU06781	β-1,3-glucanosyltransferase GEL5	β-glucan remodelling
Cell wall synthesis and remodelling	*dfg5*	NCU03770	GH76 glycosyl hydrolase DFG5	Incorporation of GPI-anchored cell wall proteins
Cell wall synthesis and remodelling	*dcw1*	NCU08127	GH76 glycosyl hydrolase DCW1	Cell wall organisation

Supplementary Table S2. Primers used for RT-qPCR analysis

Primer sequences used for RT-qPCR analysis in *Neurospora crassa*.

Gene	Forward primer (5′→3′)	Reverse primer (5′→3′)
**Reference gene**	**Reference gene**	**Reference gene**
hH3	GCGTCTCGTCCGTGAGATTG	TTAGCGTGGATAGCGCAGAG
**Ergosterol biosynthesis and sterol regulation**	**Ergosterol biosynthesis and sterol regulation**	**Ergosterol biosynthesis and sterol regulation**
cyp51	CGGTGTAAATCTCTTCGGCG	GTCCTCCAGCAGTTCCTCTT
erg3	ATTGTCAAGGAGGTGGAGGG	CATCCCACTTCGCATGAAGG
erg4	CTGAAGCCGGTAATCAAGCC	TCGTGACGACCTGGGATATG
erg5	TTTCACCTTCCTCTTCGCTTCCCA	TCATCGACTCAAGCTGCTCCATGT
erg6	GGCGGTACCTTTGGTGTCTA	ATGAGGTACATGGGGGTAAA
erg24	TACTCGCCAAGCAATACC	CTCCGAAACTCAGCATGAA
sah2	ACCGAAGACAGGGAGGAACT	GCATGCTGTTGTTCTCCTCA
**HOG signalling and osmoadaptation**	**HOG signalling and osmoadaptation**	**HOG signalling and osmoadaptation**
os-2 (hog1)	AATGATGCTGATCTCCCGGT	TGTTTTATTGCGGCGGAACA
gpd1	GAGGTTGAGAAGACGGAGCTTAA	CACCTTAATCTCCTGGTCGATGA
gpp1	CAGATTGGGTGGTGAGGGAT	TACCACAATCAACCCAGCCT
**Oxidative stress response**	**Oxidative stress response**	**Oxidative stress response**
nap-1	GATGTGAGCCGGAAAGGTTA	CTCTCCAATCGCAGAAAAGG
cat-1 (cat)	CTGCTGGGTTCTTGGGAGA	AAGTACACCAACTGCCACCA
cat-2 (pcat)	ACAAGGGCCGATCTCATCTT	TCCTGCTTGAGATCGAACCG
sod-1	CCGTGACTGACAACCTCGT	CTTGCCAAGGTCGTCAGTG
**Cell-wall synthesis and remodelling**	**Cell-wall synthesis and remodelling**	**Cell-wall synthesis and remodelling**
fks1	TGGCCACCTGATTCTGTTCT	GATCACACGTCTCCTCCTGA
ccg-8	AAGGTGGCTCTCTCCTTTA	GGTCATTTGGTTCATCTTCTTG
chs1	CGTGTCTTGGCTGTTTTCCA	GCACCAGTAGCGAACAGAAG
chs3	GCCTACGGTGTGACTCTGAT	CGATGTAGATGGCTGCGAAC
chs4	GCGATCTTTGCGGTACCTTC	GAGGAATGATTTGTGGCGGG
chs5	GGCTCTTCATACGGTTGTCC	GATCCCTTGCTCGACTTCCT
chs6	GGGTCGTCTTTGATGGTACCTA	ACATGCTATTCCCACCGGTA
chs7	CGCCAAGATTGCCGGTATTT	CGCAAGCGGTACATGGTGAA
bgt2	ATCACCGACAACCTCTCCAC	TAGCAGGCTCGGAAGAAGAG
bgt3	CTTGCAACTCTCACGCCTAC	TGACGAAACCAGGCTTGTTG
dfg5	ATGGTACTGCGAATCGGGAT	GCGAAGTTGATGAGAGCCAC
dcw1	TTATTGATGAAGCCGACCCG	CAGCCTTATCCGCCGTAGTA
gel1	TCTAACAACGCCACCAACAC	CAGTTGAAGAAGTGCGCCAT
gel3	GTGTCTACTCGGGAGGTCTT	TGGCAAATTCAGGCTTCTCC
gel5	TGACGTTCTCTTCGACAGCT	TTCACCTCGTTGCAGCCATA

Supplementary Table S3. Statistical analysis of RT-qPCR gene-expression data following azole treatment

Statistical analysis was performed using ΔCt values from three independent biological replicates. For experiments comparing fluconazole (FLC), voriconazole (VRC), and ravuconazole (RVC) with a common vehicle control (1% DMSO), differences were evaluated by one-way ANOVA followed by Dunnett's multiple-comparison test. Ketoconazole (KTC) was analysed either in the same experimental set or, where indicated, against a matched control from an independent experimental set. The table reports the overall one-way ANOVA p-value and the comparison-specific Dunnett-adjusted p-values. For experiments in which KTC was evaluated with a separate matched control, the corresponding p-value is reported in the final column. Statistical significance was defined as $p < 0.05$; significant p-values are shown in bold.

Functional group	Gene	Overall one-way ANOVA p	FLC vs control (Dunnett adjusted p)	VRC vs control (Dunnett adjusted p)	RVC vs control (Dunnett adjusted p)	KTC vs common control (Dunnett adjusted p)	KTC vs matched control p
Glycerol biosynthesis	*gpd1*	**3.738×10^{-8}**	**3.145×10^{-13}**	**1.368×10^{-8}**	**2.405×10^{-12}**	n/a	0.0931865
Glycerol biosynthesis	*gpp1*	**0.00209974**	**0.00258335**	**0.0452616**	**0.00154533**	n/a	**0.04244**
Antioxidant defence	*cat*	**3.911×10^{-10}**	**1.589×10^{-10}**	**6.661×10^{-16}**	**1.000×10^{-16}**	n/a	**0.00542337**
Antioxidant defence	*pcat*	**1.197×10^{-13}**	**1.000×10^{-16}**	**1.000×10^{-16}**	**1.000×10^{-16}**	n/a	**0.000156925**
Antioxidant defence	*sod-1*	**1.601×10^{-11}**	**1.000×10^{-16}**	**1.829×10^{-8}**	**1.000×10^{-16}**	n/a	**0.00213339**
Stress signalling / transcriptional regulation	*hog1*	**2.906×10^{-6}**	**1.005×10^{-7}**	**4.097×10^{-6}**	**9.070×10^{-8}**	n/a	**0.00178966**
Stress signalling / transcriptional regulation	*ccg-8*	**7.523×10^{-8}**	**2.319×10^{-10}**	**3.439×10^{-5}**	0.751472	n/a	**0.00234084**
Stress signalling / transcriptional regulation	*nap-1*	**0.0143939**	0.246267	0.859538	0.0841165	n/a	0.110617
Stress signalling / transcriptional regulation	*sah2*	**0.013**	0.237	0.0778	**0.0028**	0.318	n/a
Ergosterol biosynthesis	*cyp51*	**2.298×10^{-7}**	**0.00155842**	**1.601×10^{-10}**	**1.035×10^{-7}**	n/a	**0.000308724**
Ergosterol biosynthesis	*erg5*	**0.000357857**	**0.000573394**	0.995314	**0.00737404**	n/a	0.313046
Cell-wall synthesis / remodelling	*fks1*	**1.799×10^{-9}**	**1.000×10^{-16}**	**0.0438347**	**2.930×10^{-13}**	n/a	**0.00249175**
Cell-wall synthesis / remodelling	*chs1*	**6.290×10^{-9}**	**0.00143**	**6.170×10^{-9}**	**4.400×10^{-7}**	**1.000×10^{-6}**	n/a
Cell-wall synthesis / remodelling	*chs3*	**0.0157307**	0.204777	0.560298	0.596227	0.0558261	n/a
Cell-wall synthesis / remodelling	*chs4*	**0.000328571**	**0.00506148**	**0.000218872**	**0.016855**	0.943899	n/a
Cell-wall synthesis / remodelling	*chs5*	**7.676×10^{-8}**	**0.0168446**	**2.454×10^{-9}**	**0.0450164**	**0.0206128**	n/a
Cell-wall synthesis / remodelling	*chs6*	0.462023	0.49688	0.403083	0.997907	0.991218	n/a
Cell-wall synthesis / remodelling	*chs7*	**3.280×10^{-7}**	0.125767	**1.133×10^{-6}**	**0.00305054**	0.635222	n/a
Cell-wall synthesis / remodelling	*bgt2*	**0.00112171**	**0.00242413**	0.79119	0.999893	0.984044	n/a
Cell-wall synthesis / remodelling	*bgt3*	**0.000186142**	**0.000688596**	0.944365	**0.0080091**	0.526884	n/a
Cell-wall synthesis / remodelling	*dfg5*	**4.386×10^{-8}**	**0.0197271**	**1.109×10^{-10}**	0.916466	0.899708	n/a
Cell-wall synthesis / remodelling	*dcw1*	**0.000890231**	**0.0163706**	0.055203	0.866849	0.482818	n/a
Cell-wall synthesis / remodelling	*gel1*	**0.00544067**	0.0545354	**0.00940056**	0.977531	0.921957	n/a
Cell-wall synthesis / remodelling	*gel3*	**4.006×10^{-7}**	0.180922	**2.045×10^{-8}**	0.571326	0.631334	n/a
Cell-wall synthesis / remodelling	*gel5*	**0.000962643**	**0.00482901**	0.528468	0.405271	0.994613	n/a

Supplementary Table S4. Comparison of β-tubulin and histone H3 (hH3) as reference genes for RT-qPCR normalisation

The same target-gene Ct values from one representative biological replicate of control and azole-treated mycelia were normalised independently to β-tubulin or histone H3 (hH3; NCU01635). Relative expression was calculated by the $2^{-\Delta\Delta Ct}$ method, with the corresponding control set to 1. Because the comparison was based on one representative biological replicate, it is presented as a reference-gene concordance check rather than a formal multi-replicate stability analysis. Treatment-response patterns were qualitatively similar with both reference genes, and hH3 was used consistently for the complete dataset.

Treatment	Reference gene	Reference-gene Ct	gpd1	gpp1	hog1	cat	pcat	sod-1	nap-1
Control	β-tubulin	21.91	*1.000*	*1.000*	*1.000*	*1.000*	*1.000*	*1.000*	*1.000*
FLC	β-tubulin	19.12	*12.55*	*0.483*	*7.362*	*11.00*	*9.190*	*4.823*	*0.646*
VRC	β-tubulin	20.93	*4.408*	*0.655*	*5.389*	*121.1*	*5.979*	*1.659*	*0.877*
RVC	β-tubulin	19.99	*8.938*	*0.451*	*7.311*	*109.1*	*0.041*	*3.758*	*2.028*
KTC	β-tubulin	19.13	*7.160*	*0.758*	*6.589*	*15.14*	*30.91*	*2.204*	*1.329*
Control	hH3	17.90	*1.000*	*1.000*	*1.000*	*1.000*	*1.000*	*1.000*	*1.000*
FLC	hH3	15.70	*18.90*	*0.727*	*11.08*	*16.56*	*13.83*	*7.260*	*0.973*
VRC	hH3	17.10	*4.993*	*0.742*	*6.105*	*137.2*	*6.774*	*1.879*	*0.993*
RVC	hH3	15.50	*6.409*	*0.323*	*5.242*	*78.25*	*0.029*	*2.694*	*1.454*
KTC	hH3	15.30	*8.112*	*0.859*	*7.464*	*17.15*	*35.02*	*2.497*	*1.505*

Ct, threshold cycle; FLC, fluconazole; VRC, voriconazole; RVC, ravuconazole; KTC, ketoconazole.

Supplementary Table S5. Two-way ANOVA and post-hoc analysis of glycerol accumulation following azole exposure

Glycerol accumulation was analysed by two-way ANOVA with treatment (matched vehicle control, FLC, VRC, RVC, and KTC) and exposure time (2, 4, and 6 h) as independent factors. Significant effects were detected for treatment ($p = 3.22 \times 10^{-11}$), time ($p = 3.66 \times 10^{-6}$), and their interaction ($p = 3.73 \times 10^{-6}$). Dunnett's multiple-comparison test was then used to compare each azole treatment with the matched vehicle control at each time point. Data were obtained from three independent biological replicates. NS, not significant; *p < 0.05; **p < 0.01; ***p < 0.001; ****p < 0.0001.

A. Two-way ANOVA

Source	Sum of squares	df	F	p-value	Interpretation
Treatment	237.73	4	37.1546	**3.218×10^{-11}**	Significant
Time	62.5727	2	19.5589	**3.656×10^{-6}**	Significant
Treatment × Time	113.787	8	8.8918	**3.726×10^{-6}**	Significant
Residual	47.988	30	n/a	n/a	n/a

B. Dunnett-adjusted comparisons

Time (h)	Comparison	Dunnett statistic	Adjusted p-value	Significance
2	FLC vs control	4.89017	**0.0021933**	**
2	VRC vs control	6.82311	**0.000132428**	***
2	RVC vs control	7.67341	**7.335×10^{-5}**	****
2	KTC vs control	8.82101	**1.470×10^{-5}**	****
4	FLC vs control	0.627877	0.919547	NS
4	VRC vs control	-0.704619	0.886342	NS
4	RVC vs control	0.664258	0.904479	NS
4	KTC vs control	6.28075	**0.000354097**	***
6	FLC vs control	-3.67405	**0.0140108**	*
6	VRC vs control	-1.78766	0.280605	NS
6	RVC vs control	-3.05937	**0.0380425**	*
6	KTC vs control	3.90318	**0.00978801**	**
